# The Role of Fasting LDL-C Levels in Their Non-fasting Reduction in Patients With Coronary Heart Disease

**DOI:** 10.3389/fcvm.2021.686234

**Published:** 2021-06-16

**Authors:** Qiuzhen Lin, Yan Fu, XueYan Zang, Qiming Liu, Ling Liu

**Affiliations:** ^1^Department of Cardiovascular Medicine, The Second Xiangya Hospital, Central South University, Changsha, China; ^2^Research Institute of Blood Lipid and Atherosclerosis, Central South University, Changsha, China; ^3^Modern Cardiovascular Disease Clinical Technology Research Center of Hunan Province, Changsha, China; ^4^Cardiovascular Disease Research Center of Hunan Province, Changsha, China

**Keywords:** LDL-C, fasting, non-fasting, change, coronary heart disease

## Abstract

The level of low-density lipoprotein cholesterol (LDL-C) decreases to a certain extent after daily meals; however, the influencing factor of this phenomenon has not been fully elucidated. This study included 447 patients with coronary heart disease (CHD). Serum levels of blood lipid parameters at 0, 2, and 4 hours (h) after a daily breakfast were monitored in all subjects. The levels of total cholesterol (TC), LDL-C, high-density lipoprotein cholesterol (HDL-C) and non-HDL-C significantly decreased, while those of triglycerides (TG) and remnant cholesterol (RC) significantly increased from baseline to 4 h in both male and female patients (*P* < 0.05). Multiple linear regression analysis showed that fasting LDL-C level, the non-fasting change in RC level at 4 h and fasting TG level were significant predictors of the non-fasting change in LDL-C level at 4 h in patients with CHD, and fasting LDL-C level was the most significantly associated with the non-fasting change in LDL-C level. Patients with lower levels of fasting LDL-C had smaller non-fasting changes in LDL-C levels. When the fasting LDL-C level was <1.4 mmol/L, both absolute reduction and percent reduction in LDL-C level at 4 h were almost zero, which means that the non-fasting LDL-C level at 4 h was approximately equivalent to its fasting value (*P* < 0.05). This result indicated that the non-fasting changes in LDL-C levels were influenced by fasting LDL-C levels in patients with CHD. When the fasting LDL-C level was <1.4 mmol/L, the non-fasting LDL-C level could replace the fasting value to guide treatment.

## Introduction

Commonly, it is believed that an elevated level of low-density lipoprotein cholesterol (LDL-C) in the fasting state is an important factor predicting coronary heart disease (CHD). However, most humans are in a non-fasting or postprandial state because a fasting state only occurs a few hours before breakfast (1–3). Non-fasting detection of blood lipids has been recommended in clinical practice (4–8). Therefore, increasing attention has been given to the relationship between non-fasting lipid levels and CHD (8–10). It was demonstrated that associations of non-fasting lipid levels [e.g., non-fasting LDL-C and triglycerides (TG)] with coronary events were similar to those for fasting lipid levels (11–13).

Postprandial hypertriglyceridemia presents an increase in TG-rich lipoproteins (TRLs) in the blood. Cholesterol, but not TGs, within TRL remnants plays a more important role in atherosclerosis. Moreover, the former is termed remnant cholesterol (RC) and can be estimated with the recommended formula (14–16).

In contrast to the increased level of TGs or RC after a meal, LDL-C levels decreased to a certain extent after a daily meal (1, 2, 8, 11, 17, 18). According to the consensus statement issued by the European Atherosclerosis Society and the European Federation of Clinical Chemistry and Laboratory Medicine (EAS/EFLM), non-fasting LDL-C levels only slightly change [i.e., decline by 0.2 mmol/L after habitual meals for 1–6 hours (h)] (8). However, our previous study showed that the non-fasting decrease in LDL-C levels was ~0.55 mmol/L when directly measured by enzymatic determination or 0.40 mmol/L when calculated by the Friedewald formula (19). It seems that the non-fasting decrease in LDL-C levels in Chinese subjects could be greater than that in European subjects.

However, the underlying mechanism of the non-fasting decline in LDL-C levels has not been fully elucidated. One explanation might be fluid intake and subsequent blood dilution (1, 8, 17). However, it cannot fully explain the decline in LDL-C levels in Chinese subjects after a daily meal, and there may be other unknown causes. To date, studies involving the potential causes of the decrease in LDL-C levels after a daily meal are very rare (1, 8), especially in Chinese patients with CHD. For this purpose, we explored the possible factors influencing the decrease in LDL-C levels after a daily breakfast from the perspective of blood lipids in this study.

## Materials and Methods

### Study Population

From August 2015 to October 2019, a total of 447 inpatients with CHD at the Department of Cardiovascular Medicine in the Second Xiangya Hospital of Central South University were enrolled in our study. CHD was defined as a history of myocardial infarction (MI) and/or angiographically proven coronary atherosclerosis in patients with angina pectoris.

To clearly analyze the role of fasting LDL-C level in its non-fasting reduction, according to the European dyslipidaemias management guidelines of 2019, the goal LDL-C level for patients with CHD decreased from <1.8 mmol/L to <1.4 mmol/L (20). Patients were divided into three subgroups with fasting LDL-C levels > 1.8 mmol/L (subgroup 1), 1.4–1.8 mmol/L (subgroup 2) and <1.4 mmol/L (subgroup 3).

This study was approved by the Ethics Committee of the Second Xiangya Hospital of Central South University and conformed to the 1975 Declaration of Helsinki. Informed consent was gained from all subjects.

### Specimen Collection

After fasting for at least 8 h, venous blood samples were collected in all persons at 0, 2, and 4 h after a daily breakfast, and the tests were completed on the same day. Peripheral blood samples were obtained from the brachial veins of patients when lying down. Serum levels of total cholesterol (TC) and TGs were measured by automated enzymatic assays, and those of LDL-C and high-density lipoprotein cholesterol (HDL-C) were measured by a commercially available direct method (Wako, Japan) [i.e., selective protection method and antibody blocking method, respectively, on a HITACHI 7170A analyzer (Instrument Hitachi Ltd., Tokyo, Japan)] (19) by a technician who was blinded to the blood samples. RC = TC − (HDL-C) − (LDL-C). Non-HDL-C = TC − (HDL-C). During the 4-h period, the subjects could drink only water and walk slowly until the last blood sample was collected.

### Statistical Analysis

Statistical analysis was performed on SPSS 20.0. Data drawing was completed by GraphPad Prism 6.0 software. Quantitative variables are expressed as means ± standard deviations, and qualitative variables are expressed as numbers and percentages. Fasting TG levels not conforming to a normal distribution were log-transformed for comparison and presented as medians with 25th and 75th percentiles. Differences between the intra- and intergroup means were analyzed by Student's *t*-test or one-way ANOVA. If they found significant differences among the subgroups, a multiple comparison test was performed. Categorical variables were compared using chi-square statistic tests. Coefficients of correlation (*r*) were calculated by Pearson correlation analysis. An association between the changed level of LDL-C and its risk factors was determined by linear regression analysis. All *P*-values were 2-tailed, and *P* < 0.05 was considered statistically significant.

## Results

### Clinical Characteristics and Fasting Blood Lipids

Body mass index (BMI), the percentages of smokers and the number of risk factors were significantly higher in male patients with CHD (*P* < 0.05). There was no significant difference in the levels of TGs, TC, LDL-C, non-HDL-C and RC, except for HDL-C, between male and female patients (Table 1).

**Table 1:** Clinical characteristics of patients with CHD between male and female.

	**Male (*n* = 326)**	**Female (*n* = 121)**	***P*-value**
Age, y	59.4 ± 10.3	64.5 ± 9.4	<0.05
BMI, kg/m^2^	25.2 ± 3.4	24.1 ± 4.8	<0.05
Smoking, *n* (%)	227 (69.9)	7 (5.8)	<0.05
Hypertension, *n* (%)	237 (72.7)	96 (79.3)	ns
Diabetes, *n* (%)	87 (26.7)	42 (34.7)	ns
Number of risk factors^*^ per capita, *n*	2.0 ± 1.0	1.4 ± 1.0	<0.05
Statins using^#^, *n* (%)			ns
None	104 (31.9)	42 (34.7)	
<1 month	86 (26.4)	25 (20.7)	
1 month or more	136 (41.7)	54 (44.6)	
**Fasting blood lipids**			
TG, mmol/L	1.45 (1.10, 2.15)	1.45 (1.05, 2.10)	ns
TC, mmol/L	3.82 ± 0.93	3.95 ± 1.13	ns
LDL-C, mmol/L	2.38 ± 0.82	2.38 ± 1.00	ns
HDL-C, mmol/L	0.97 ± 0.23	1.07 ± 0.29	<0.05
RC, mmol/L	0.46 ± 0.24	0.50 ± 0.28	ns
Non-HDL-C, mmol/L	2.85 ± 0.87	2.87 ± 1.12	ns

*Data were expressed as mean ± standard deviation, median (25th−75th percentile), or n (%). CHD, coronary heart disease; BMI, body mass index; TG, Triglyceride; TC, Total cholesterol; LDL-C, LDL cholesterol; HDL-C, HDL cholesterol; RC, Remnant lipoprotein cholesterol; non-HDL-C, non-HDL cholesterol*.

* *Risk factors including smoking, hypertension, overweight, and obesity (BMI ≥ 24 kg/m^2^) and diabetes*.

# *Taking statins before admission*.

### Non-fasting Changes in Blood Lipid Levels After a Daily Meal

**Figure 1:**
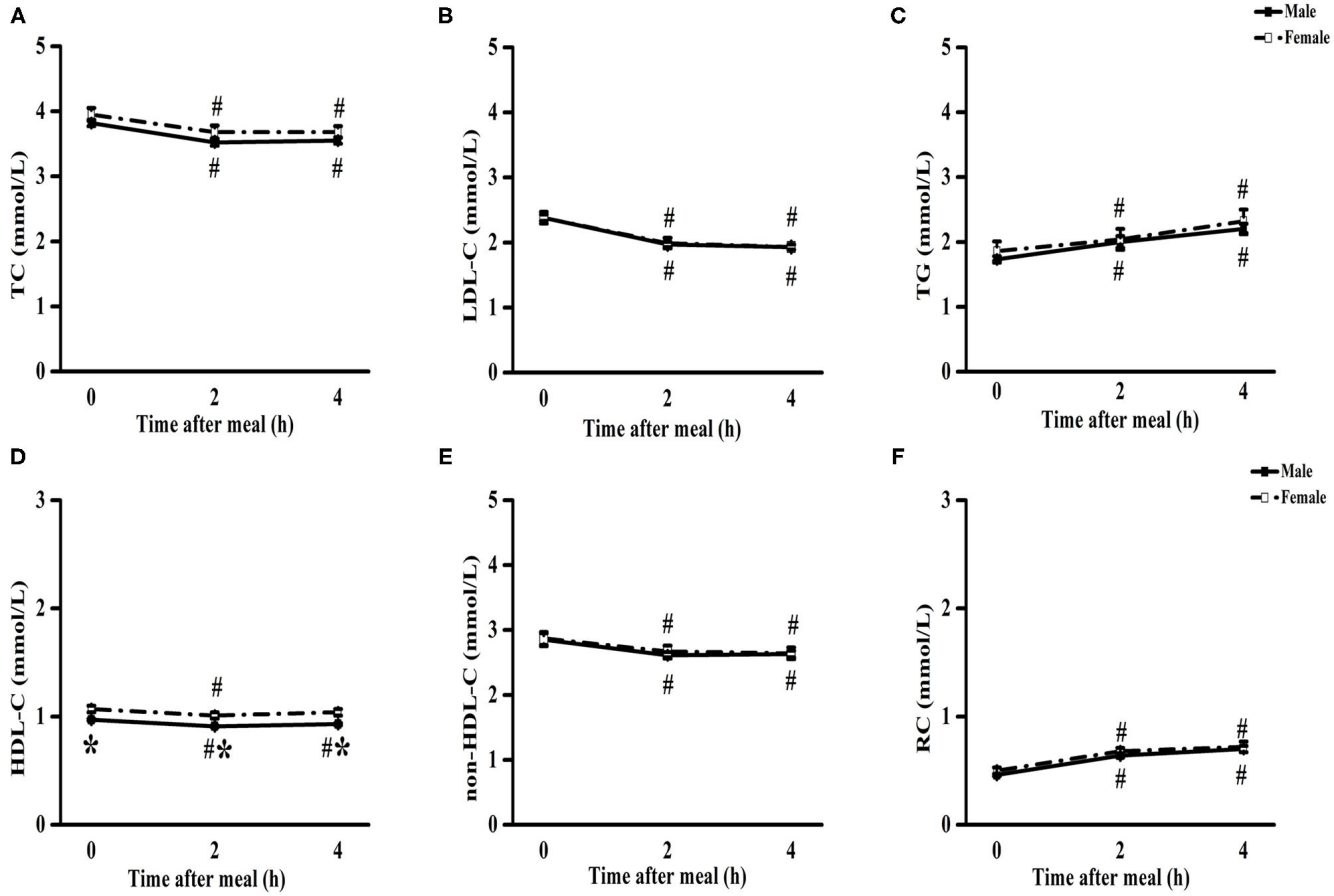
Comparisons of blood lipids in patients with CHD between male and female after a daily breakfast. Comparisons of TC **(A)**, LDL-C **(B)**, TG **(C)**, HDL-C **(D)**, non-HDL-C **(E)** and RC **(F)** levels between male and female patients with CHD. ^#^*P* < 0.05 when compared with fasting value within the same group, *P < 0.05 when compared with those of female patients at the same time point.

The levels of TC, LDL-C, HDL-C, and non-HDL-C significantly decreased, while those of TGs and RC significantly increased from baseline to 4 h in male and female patients (*P* < 0.05) (Figure 1). There was no significant difference in the levels of TC, LDL-C, TGs, non-HDL-C, and RC between male and female patients at each non-fasting time point (Figures 1A–C,E,F). However, the levels of HDL-C were significantly lower in male patients compare to that in female patients at each time point (*P* < 0.05) (Figure 1D).

### Analysis of Factors Associated With Non-fasting Changes in LDL-C Levels

Pearson's correlation analysis showed that the non-fasting change in LDL-C level at 4 h was negatively correlated with the fasting LDL-C level (*r* = −0.512; *P* < 0.05), non-fasting change in RC level at 4 h (*r* = −0.474; *P* < 0.05) and fasting TG level (*r* = −0.354; *P* < 0.05) in patients with CHD (Supplementary Table 2).

Multiple linear regression analysis showed that the fasting LDL-C level, non-fasting change in RC level at 4 h and fasting TG level were significant predictors of the non-fasting change in LDL-C level at 4 h in patients with CHD, all of which explained 46.5% of the non-fasting change in the LDL-C level (*P* < 0.05) (Supplementary Table 3). More importantly, the fasting LDL-C level (regression coefficient β was the highest in each model) was most significantly associated with the non-fasting change in LDL-C level at 4 h (Supplementary Table 3).

### Fasting Blood Lipids in Three Subgroups

It is known that the LDL-C goal level in patients with CHD decreased from <1.8 mmol/L to <1.4 mmol/L. Thus, patients with CHD were divided into three subgroups with fasting LDL-C levels > 1.8 mmol/L (subgroup 1), 1.4–1.8 mmol/L (subgroup 2) and <1.4 mmol/L (subgroup 3). The highest fasting TG level was found in subgroup 1 (*P* < 0.05), and the lowest fasting levels of TC, LDL-C, and non-HDL-C were found in subgroup 3, which was associated with a high rate and long duration of statin treatment (*P* < 0.05) (Supplementary Table 1).

### Comparison of the Non-fasting Changes in Blood Lipids Among Three Subgroups

**Figure 2:**
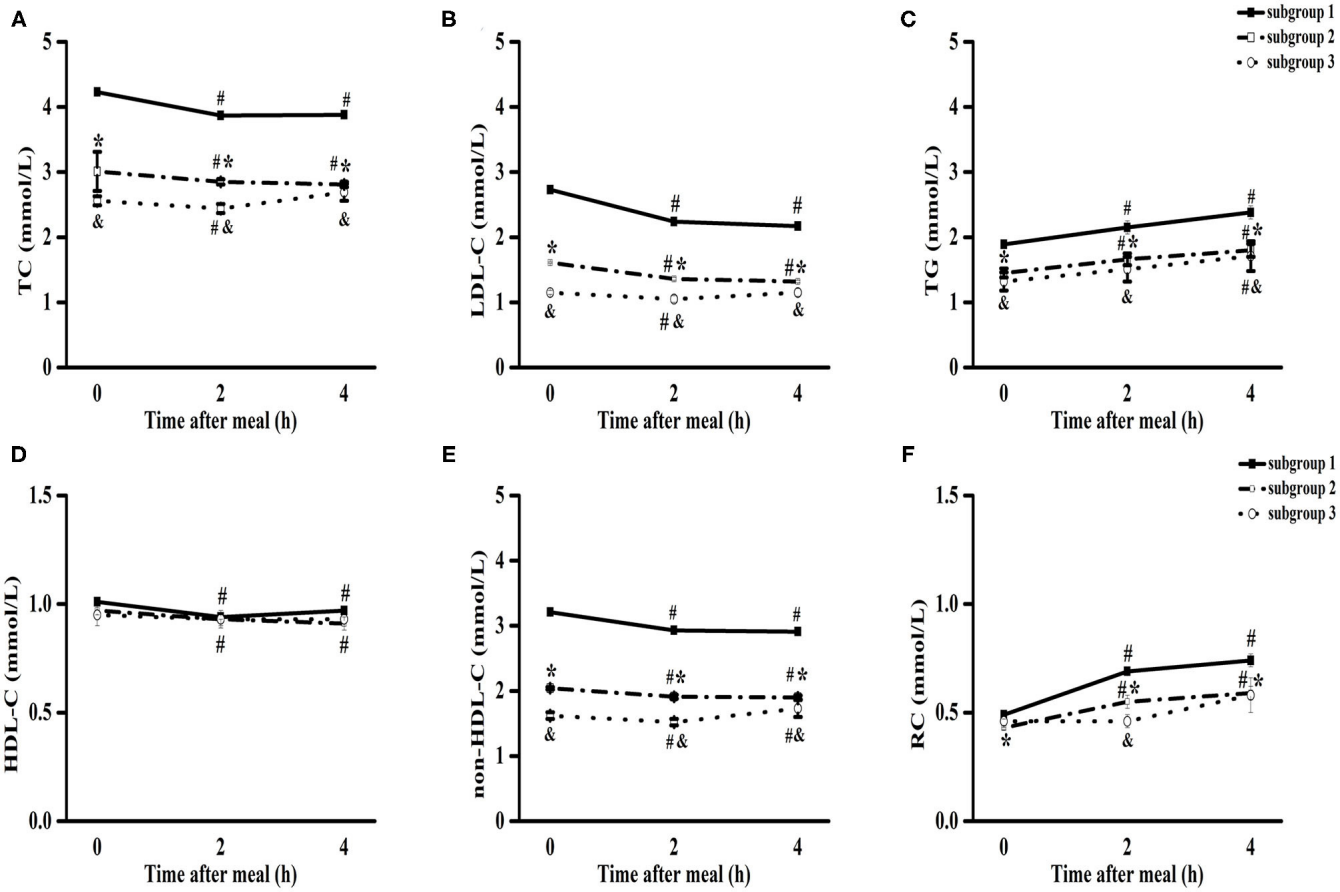
Comparisons of the blood lipids levels among three subgroups of patients with CHD after a daily breakfast. Comparisons of TC **(A)**, LDL-C **(B)**, TG **(C)**, HDL-C **(D)**, non-HDL-C **(E)** and RC **(F)** levels among three subgroups after a daily breakfast. ^#^*P* < 0.05 when compared with fasting value within the same subgroup, *P < 0.05 when the subgroup 2 compared with the subgroup 1 at the same time point, ^&^*P* < 0.05 when the subgroup 3 compared with the subgroup 1 at the same time point, ^#^ in **(D)** meant non-fasting HDL-C values were significantly lower than those of fasting values in subgroup 1 and subgroup 2.

The levels of TC, LDL-C, HDL-C, and non-HDL-C significantly decreased, while those of TG and RC significantly increased from baseline to 4 h in subgroup 1 and subgroup 2 (*P* < 0.05) (Figure 2). However, there was no significant difference in the levels of TC, LDL-C, HDL-C, and RC between fasting and non-fasting states (4 h) in subgroup 3 (Figure 2).

**Figure 3:**
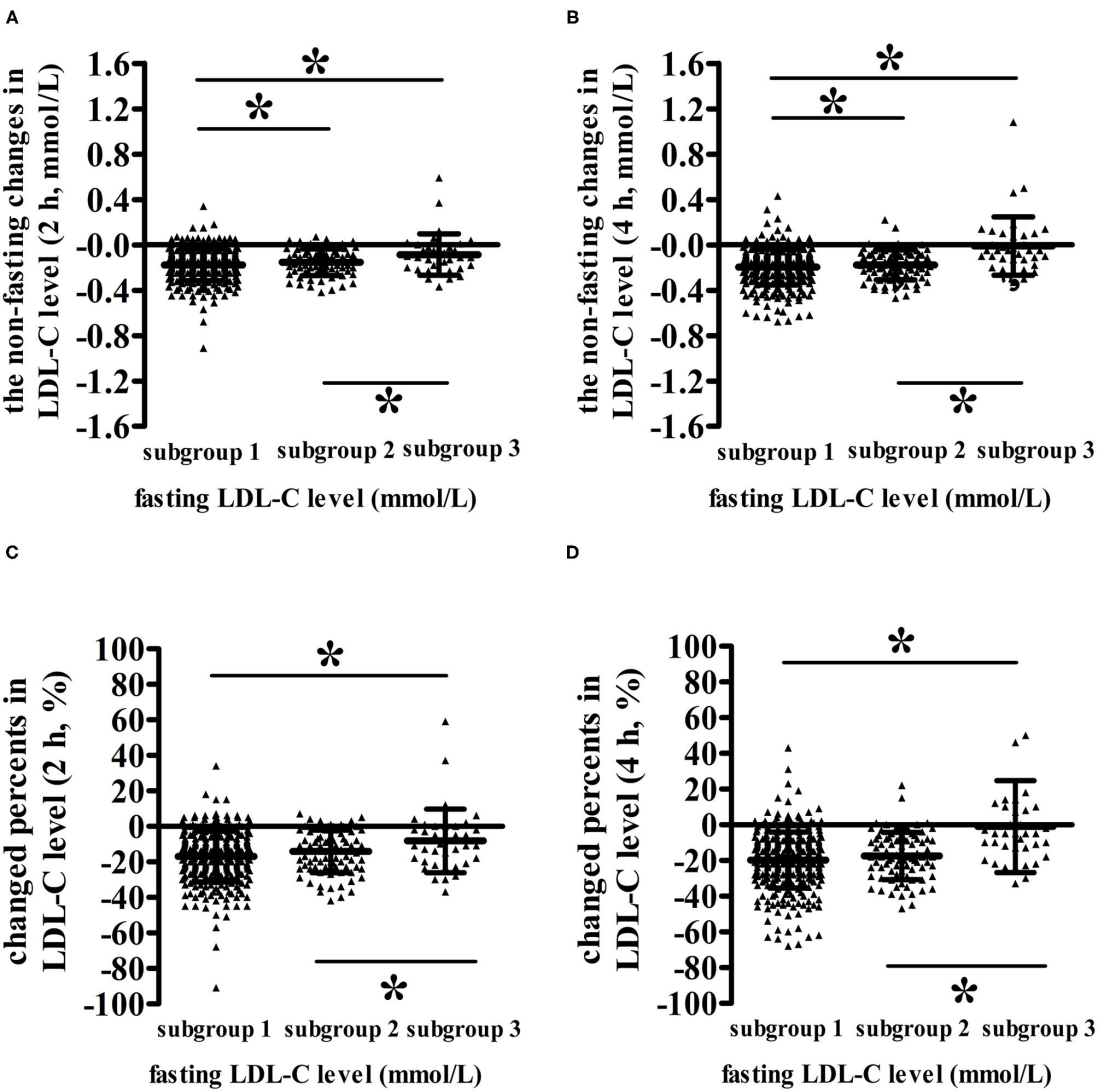
Comparisons of the non-fasting changes and changed percents in LDL-C levels among patients with different fasting LDL-C levels after a daily breakfast. **(A,B)** Comparisons of the non-fasting changes of LDL-C levels among patients with different fasting LDL-C levels at 2 and 4 h, respectively. **(C,D)** Comparisons of the non-fasting changed percents of LDL-C levels among patients with different fasting LDL-C levels at 2 and 4 h, respectively. *P < 0.05 the difference between two subgroups reached statistical significance.

The lower the fasting LDL-C level was, the smaller the non-fasting change in LDL-C level (Figure 3). When the fasting LDL-C level was <1.4 mmol/L, both absolute reduction (the changed level was −0.004) and percent reduction (the changed percent was −0.93%) in LDL-C level at 4 h were almost zero, indicating that the non-fasting LDL-C level at 4 h was approximately equivalent to its fasting value in subgroup 3 (*P* < 0.05) (Figures 3B,D). The absolute reduction (the changed level was −0.099) and percent reduction (the changed percent was −8.57%) in LDL-C levels at 2 h were also approximately equivalent to zero (*P* < 0.05) (Figures 3A,C).

## Discussion

To our knowledge, this is the first study to analyze the effect of fasting LDL-C levels on changes in LDL-C levels in Chinese subjects after a daily meal. Non-fasting LDL-C levels decreased obviously when fasting LDL-C levels were above 1.4 mmol/L, especially at 4 h after a daily breakfast. There were three lipid parameters that significantly contributed to the non-fasting change in LDL-C level at 4 h in this study, including the fasting LDL-C level, change in RC level at 4 h and fasting TG level. The lower the fasting LDL-C level was, the smaller the non-fasting change in LDL-C level in patients with CHD. Once the fasting LDL-C level was lower than 1.4 mmol/L, the non-fasting LDL-C level at 4 h was approximately equivalent to its fasting value. This result indicated that the non-fasting detection of blood lipids could substitute for the fasting detection during the follow-up, as long as the fasting LDL-C level of patients with CHD was low enough (i.e., <1.4 mmol/L).

Although fasting and non-fasting LDL-C levels showed similar predictive values for all-cause death and cardiovascular death (12), clinicians still have some doubts about non-fasting detection of blood lipids. This was because the correlation between fasting or non-fasting blood lipids and cardiovascular risk was assessed by measuring blood lipids in different individuals in the fasting or non-fasting state rather than in the same individuals in some important studies (1, 17). Therefore, when clinicians evaluate the cardiovascular risk of a specific individual, they cannot identify the difference in fasting and non-fasting lipid levels. Recently, some researchers compared the predictive value of fasting and non-fasting lipid levels on cardiovascular events in the same individuals in a prospective study (11). The results showed that the fasting and non-fasting lipid level measurements yielded similar results related to cardiovascular events in the same individual. Notably, there was a 4-week interval between those two tests in that study, which is different from the multiple tests of blood lipids within 1 day in this study. Additionally, Langston et al. (1, 17) attributed the decrease in LDL-C level after a daily meal to blood dilution by fluid intake. Moreover, the assay kit or method (21), sample size (21–23), underlying diseases (24), race and dietary habits of subjects could also affect the change in LDL-C level after a meal (1, 17, 25, 26). However, few researchers have explored the effects of lipid factors on non-fasting LDL-C reduction.

The change in LDL-C level after a daily meal was completely opposite to that in TG or RC level, indicating there may be some connection between them. Although TGs mainly exist in TRLs, the RC level estimated by the formula can also reflect the particle numbers of TRLs and their remnants in the circulation, similar to the TG level (3). This could explain the close relationship between RC levels and TG levels in a non-fasting state (15). Non-fasting RC levels not only include cholesterol in very low density lipoprotein (VLDL) and its remnants and intermediate density lipoprotein (IDL) but also contain cholesterol in chylomicrons (CMs) in the non-fasting state (16, 27, 28). CMs are not only the most TRLs but also the main ones to transport dietary cholesterol in the circulation. Under normal conditions, the TGs in TRLs will be rapidly hydrolyzed by lipoprotein lipase (LPL) located on the surface of capillaries. In this study, the increase in RC level at 4 h and fasting TG level were significantly correlated with the changes in LDL-C level at 4 h after a meal, which was similar to the finding of another study (29). One possible explanation was that increased TRLs and their remnants could activate cholesteryl ester transporter protein (CETP), promoting the transfer of cholesteryl ester from HDL and LDL to TRLs and their remnants and the reverse transfer of TGs among those particles, ultimately leading to increased RC levels and decreased HDL-C and LDL-C levels in the non-fasting state (30, 31). It has been demonstrated that dietary cholesterol can upregulate CETP synthesis (32), which partly supports the former opinion.

More importantly, the effect of fasting LDL-C levels on postprandial changes in LDL-C levels should not be ignored. There was a positive correlation between CETP activity and fasting LDL-C levels in children with type 1 diabetes and obesity (33). Moreover, adult patients with hypercholesterolemia presented an increased transport rate of CETP (34, 35). It is worth noting that the quality of plasma CETP is highly relevant to its activity (36, 37). It was reported that fasting LDL-C levels were positively related to plasma CETP mass in patients with type 2 diabetes treated with atorvastatin (38). Consequently, it is reasonable to assume that fasting LDL-C levels could play an exact role in the non-fasting decline of LDL-C levels by influencing the activity and/or mass of CETP. The lower the fasting LDL-C level was, the lower the transport rate of CETP, resulting in a smaller non-fasting decline in LDL-C levels. In addition, the non-fasting decrease in LDL-C levels after a daily breakfast may be influenced by the circadian rhythm of liver cholesterol synthesis. We noticed that TC and LDL-C levels decreased when the fasting LDL-C level was above 1.4 mmol/L after a daily breakfast and reached the lowest level at 4 h, which is consistent with the circadian rhythm of liver cholesterol synthesis, that is, the highest at midnight and the lowest at noon (39). Compared with the daily low-fat and low-calorie diet, the high-fat and high-calorie diet is more likely to disturb the circadian rhythm of cholesterol synthesis, resulting in a slight increase in non-fasting TC levels (40).

According to the European dyslipidaemias management guidelines of 2019, the goal LDL-C level for patients with CHD decrease from <1.8 mmol/L to <1.4 mmol/L, in addition to a >50% reduction from baseline (20). Compared with patients with fasting LDL-C > 1.8 mmol/L, the decrease in LDL-C at 4 h after a meal was not obvious in those with a fasting LDL-C <1.4 mmol/L. Skoczyńska et al. (24) found that non-fasting LDL-C levels in patients with metabolic syndrome decreased more obviously than those in healthy individuals and conjectured that the non-fasting decline in LDL-C levels could be associated with increased cholesterol influx into the blood vessel wall; thus, patients with this feature were more likely to develop atherosclerosis. Of course, this view needs to be further explored and verified. Our findings suggested that, for patients whose fasting LDL-C levels were low enough, non-fasting LDL-C levels at 4 h after a daily meal could be close to its fasting value, which increased the feasibility of detecting non-fasting blood lipids in patients with CHD.

## Conclusions

This study showed that non-fasting changes in LDL-C levels were influenced by fasting LDL-C levels in Chinese patients with CHD. More importantly, when the fasting LDL-C level was <1.4 mmol/L, the non-fasting LDL-C level could replace the fasting value to guide treatment.

## Limitations

There were several limitations to this study. First, the number of female subjects was relatively small. Second, this was a cross-sectional study, not a prospective study. Third, the number of subjects with a fasting LDL-C <1.4 mmol/L was relatively small, which reflected the actual situation of hospitalized patients with CHD. Finally, a prospective study with a large population needs further exploration in the future.

## Data Availability Statement

The original contributions presented in the study are included in the article/Supplementary Material, further inquiries can be directed to the corresponding author/s.

## Ethics Statement

The studies involving human participants were reviewed and approved by the Ethics Committee of the Second Xiangya Hospital of Central South University. The patients/participants provided their written informed consent to participate in this study.

## Author Contributions

QLin, YF, and LL designed and conducted of this study. QLin, YF, and XZ participated in the collection and analysis of the data, and contributed to the preparation of the manuscript. LL and QLiu conducted the literature review. All authors read the study and approved the manuscript for publication.

## Conflict of Interest

The authors declare that the research was conducted in the absence of any commercial or financial relationships that could be construed as a potential conflict of interest.
