# Supplementary material for: The Role of Fasting LDL-C Levels in Their Non-fasting Reduction in Patients With Coronary Heart Disease
Source: Front Cardiovasc Med. 2021 Jun 16;8:686234. doi: 10.3389/fcvm.2021.686234 (PMC8241924; doi:10.3389/fcvm.2021.686234)
Supplement: Supplementary file 1 [file Data_Sheet_1.docx]

**Supplement Table 1** Clinical characteristics of three subgroups in patients with CHD.

|  | Subgroup 1  (n = 322) | Subgroup 2  (n = 86) | Subgroup 3  (n = 39) | *P*-value |
| --- | --- | --- | --- | --- |
| Age, y | 60.0 ± 10.4 | 62.0 ± 8.8 | 63.4 ± 11.2 | ns |
| Gender, M/F | 237/85 | 63/23 | 27/12 | ns |
| BMI, kg/m^2^ | 25.1 ± 3.9 | 24.8 ± 3.0 | 24.0 ± 3.3 | ns |
| Smoking, n (%) | 174 (54.0) | 42 (48.8) | 16 (41.0) | ns |
| Hypertension, n (%) | 240 (74.5) | 62 (72.1) | 26 (66.7) | ns |
| Diabetes, n (%) | 82 (25.5) | 28 (32.6) | 18 (46.2) | < 0.05 |
| Statins using*, n (%) |  |  |  | < 0.05 |
| None | 128 (39.8) | 15 (17.4) | 8 (20.5) |  |
| Less than 1 month | 78 (24.2) | 25 (29.1) | 6 (15.4) |  |
| 1 month or more | 116 (36.0) | 46 (53.5) | 25 (64.1) |  |
| Fasting blood lipids |  |  |  |  |
| TG, mmol/L | 1.89 ± 1.27 | 1.45 ± 0.67^a^ | 1.32 ± 0.85^b^ | < 0.05 |
| TC, mmol/L | 4.23 ± 0.89 | 3.01 ± 0.27^a^ | 2.56 ± 0.42^b,c^ | < 0.05 |
| LDL-C, mmol/L | 2.73 ± 0.76 | 1.61 ± 0.12^a^ | 1.15 ± 0.18^b,c^ | < 0.05 |
| HDL-C, mmol/L | 1.01 ± 0.26 | 0.97 ± 0.22 | 0.95 ± 0.23 | ns |
| RC, mmol/L | 0.49 ± 0.28 | 0.43 ± 0.11 | 0.46 ± 0.21 | ns |
| non-HDL-C, mmol/L | 3.21 ± 0.85 | 2.04 ± 0.16^a^ | 1.62 ± 0.32^b,c^ | < 0.05 |

Note: Data were expressed as mean ± standard deviation, median (25th -75th percentile), or n (%). Abbreviation: Subgroup 1: the subgroup with a fasting LDL-C > 1.8 mmol/L; subgroup 2: the subgroup with a fasting LDL-C 1.4 - 1.8 mmol/L; subgroup 3: the subgroup with a fasting LDL-C < 1.4 mmol/L; BMI: body mass index; TG: Triglyceride; TC: Total cholesterol; LDL-C: LDL cholesterol; HDL-C: HDL cholesterol; RC: Remnant lipoprotein cholesterol; non-HDL-C: non-HDL cholesterol. * Taking statins before admission. ^a^ *P* < 0.05 when the subgroup 2 compared with the subgroup 1. ^b^ *P* < 0.05 when the subgroup 3 compared with the subgroup 1. ^c^ *P* < 0.05 when the subgroup 3 compared with the subgroup 2.

**Supplement Table 2** Pearson’s correlation coefficient between fasting LDL-C levels, the changed level of RC at 4 h, fasting TG levels and the changed level of LD-C.

| changed level of LDL-C | changed level of RC (4h) |  | LDL-C level in fasting |  | TG level in fasting |
| --- | --- | --- | --- | --- | --- |
|  | -0.474 |  | -0.512 |  | -0.354 |

**Supplement Table 3** Multiple linear regression analysis of factors associated with the non-fasting changes in LDL-C level at 4 h in patients (n = 447).

|  | Model 1 | |  | Model 2 | |  | Model 3 | |
| --- | --- | --- | --- | --- | --- | --- | --- | --- |
|  | *β* | *R^2^* |  | *β* | *R^2^* |  | *β* | *R^2^* |
|  |  | 0.340 |  |  | 0.455 |  |  | 0.465 |
| LDL-C level in fasting | -0.584 |  |  | -0.498 |  |  | -0.469 |  |
| Increase in RC level at 4 h |  |  |  | -0.352 |  |  | -0.348 |  |
| TG level in fasting |  |  |  |  |  |  | -0.110 |  |

Multiple linear regression analysis was conducted with the non-fasting changes in LDL-C level at 4 h as the dependent variable, and with fasting LDL-C level, the non-fasting changes in RC level at 4 h, fasting TG level as the independent variables. Model 1: independent variable included fasting LDL-C level; Model 2: as in model 1 further added to the non-fasting changes in RC level at 4 h; Model 3: as in model 2 further added to the fasting TG level.
